# “You did incredibly well!”: teachers’ inflated praise can make children from low-SES backgrounds seem less smart (but more hardworking)

**DOI:** 10.1038/s41539-023-00183-w

**Published:** 2023-09-01

**Authors:** Emiel Schoneveld, Eddie Brummelman

**Affiliations:** Research Institute of Child Development and Education, University of Amsterdam, Amsterdam, The Netherlands

**Keywords:** Human behaviour, Education

## Abstract

Can teachers’ inflated praise make children from low socioeconomic status (SES) backgrounds seem less smart? We conducted two preregistered experiments to address this question. We used hypothetical scenarios to ensure experimental control. An experiment with primary school teachers (*N* = 106, ages 21–63) showed that when a child from a low-SES (vs. high-SES) background succeeded in school, teachers attributed this success more to hard work and delivered more inflated praise (e.g., “You did incredibly well!”) but less modest praise (e.g., “You did well!”). An experiment with primary school children (*N* = 63, ages 10–13) showed that when children learned that another child received inflated praise (while an equally performing classmate received modest praise or no praise), they perceived this child as less smart but more hardworking. These studies provide converging evidence that teachers’ inflated praise, although well-intentioned, can make children from low-SES backgrounds seem less smart, thereby reinforcing negative stereotypes about these children’s academic abilities.

## Introduction

Achievement inequality is a pressing societal problem. Around the world, children from low socioeconomic status (SES) backgrounds are far more likely to underperform in school than their high-SES peers^1,2^, even when they have the same level of ability^3^. Teachers are motivated to combat these inequalities^4,5^. We theorize, however, that teachers may sometimes engage in well-intentioned practices that inadvertently reinforce existing inequalities. Specifically, teachers are often encouraged to lavish children from low-SES backgrounds with inflated praise. For example, when asked how to instill self-confidence in children who are stigmatized by the larger society, teachers often mention praise^6^. Although well-intentioned, inflated praise may convey to children that their success is the product of extraordinary effort rather than ability. We hypothesized that teachers would be inclined to give inflated praise to children from low-SES backgrounds, and that children would interpret the inflated praise as a sign of low ability. We report two preregistered experiments with teachers (Study 1) and children (Study 2) to test these hypotheses.

Teachers are often encouraged to praise their students^7^. Conventional wisdom holds that praising children raises their self-esteem and motivation^8–10^. Unsurprisingly, then, scholars often assume that good teachers praise frequently^11–13^, and many praise interventions aim to increase the frequency of teacher praise^14,15^. However, praise comes in different shapes and sizes. While most praise is modest, ~25% of praise is inflated^16,17^. Instead of telling students that they did well, teachers might tell them that they did incredibly well. Instead of telling students that their achievement is good, teachers might tell them that their achievement is amazing. Teachers may give inflated praise when they see a student’s success as particularly praiseworthy.

When are teachers most likely to see success as particularly praiseworthy and dole out inflated praise? Teachers’ praise is informed by their attributions of students’ success^18,19^. According to attribution theory, attributions tend to reflect three dimension: locus (internal, external), stability (stable, unstable), and controllability (controllable, uncontrollable)^18,20^. In the achievement domain, ability and effort are generally the most dominant perceived causes of success^21^. Ability and effort are both internal, but ability is considered stable and uncontrollable (e.g., intelligence, aptitude, skill, talent), whereas effort is considered unstable and controllable (e.g., hard work, perseverance, dedication). Ability and effort are typically seen as compensatory: High effort compensates for low ability, and high ability compensates for low effort^22,23^. While teachers are unlikely to praise a student when they attribute their success to external factors (e.g., luck, help from others), they are likely to praise a student when they attribute their success to internal factors (e.g., ability, effort). Like most people, teachers find effort particularly praiseworthy^24^. In prior work, for example, teachers gave the most rewards (e.g., gold stars) to students with low ability who tried hard and performed well, and gave the most punishments (e.g., red stars) to students with high ability who did not try hard and performed poorly^25^. They rewarded students with low ability because they assumed that these students had to work exceptionally hard to compensate for their lack of ability^26^. If inflated praise signals even greater praiseworthiness than does modest praise, teachers might be more inclined to give inflated praise when they believe a student succeeded through high effort. Thus, teachers’ inflated praise might track students’ presumed effort.

Building on attribution theory, we theorize that teachers give more inflated praise to children from low-SES (vs. high-SES) backgrounds, because they are more inclined to attribute low-SES children’s successes to hard work. There is a pervasive negative stereotype about the academic abilities of low-SES children^27^. Low-SES individuals are often perceived as incompetent—as “stupid,” “uneducated,” “lazy,” “unmotivated,” and “weak”^28^. In a study across 27 countries, low-SES individuals were seen as less competent than high-SES individuals^29^. In one study^30^, participants watched a video of a child taking an academic test. Participants who were told that the child came from a high-SES background rated the child’s abilities in liberal arts, reading, and mathematics *above* grade level, whereas those who were told that the child came from a low-SES background rated the same abilities *below* grade level. Even teachers hold these beliefs, as they systematically underestimate the academic abilities of low-SES children^31,32^. Unknowingly and unintentionally, teachers may be guided by these negative stereotypes. Consequently, when they see a low-SES student succeed, they may be inclined to attribute the student’s success to hard work, thus giving inflated praise.

Do teachers indeed give more inflated praise to children from low-SES backgrounds? Although this has not been investigated directly, studies have revealed a *positive feedback bias*, with White majority teachers giving more positive feedback to ethnic minority (vs. majority) students. White American evaluators gave more praise and less criticism to Black students than to White students^33–37^. White Canadian evaluators criticized Aboriginal students less than White students but praised them equally often^38^. German teachers gave students with a Turkish migration background more positive comments than they gave students without an immigration background^39^. In some cases, German teachers gave more extremely positive feedback (e.g., “I’m speechless”) to students from immigration backgrounds than to students without an immigration background^40^. However, no studies have examined such biases in the context of SES. We examined whether teachers provide more inflated praise to children from low-SES backgrounds.

Teachers’ inflated praise may, in some cases, make children appear less smart to others. Why? Children may know that teachers praise those who work hard. When two students achieve the same outcome (e.g., a high score on a test) but only one of them receives praise, children may infer that the praised student worked harder to achieve the same outcome. Consequently, they may see the praised student as less smart^41–43^. This effect may be more pronounced for inflated praise, as this type of praise is even more positive than is modest praise. Hence, when a student receives inflated praise while an equally performing student receives modest praise, the student receiving inflated praise may be perceived as less smart but more hardworking.

Although no study has investigated this idea directly, there is supportive evidence. One study involving German children^42^ and two studies involving American children^41,43^ showed that when children saw two students achieving the same outcome, they considered the student who was praised by the teacher (e.g., “Great!”) as less smart but more hardworking than the student who was not praised. This effect emerged only from late childhood, from about age 10, when children acquire the understanding that ability and effort can be compensatory. Up to age 10, children generally assume that hardworking children are smart^44^. Older children understand that if two students achieve the same outcome, the student who worked harder must be less smart^22^. However, no research has examined the effects of inflated praise on children’s perceptions of who is smart or hardworking. We examined whether children perceive students receiving inflated praise as less smart but more hardworking.

We conducted two preregistered experiments to investigate whether and how teachers may inadvertently make low-SES children seem less smart by lavishing them with inflated praise. Specifically, we investigated whether teachers provide more inflated praise to children from low-SES backgrounds (Study 1) and whether children perceive students receiving inflated praise as less smart but more hardworking (Study 2). Our study design, hypotheses, and data-analysis plans were preregistered via OSF (Study 1: https://osf.io/wka4c/; Study 2: https://osf.io/rk9q2/). We focused on children ages 10–12 years, who have developed the belief that ability and effort are compensatory^22^.

## Results

### Study 1

Study 1 investigated whether teachers provide more inflated praise to low-SES students than to equally performing high-SES students. The study had a within-subjects experimental design and was conducted online. Participants were 106 primary school teachers (ages 21–63) and provided informed consent. Teachers read four vignettes about hypothetical students. Each student was described as coming from a high-SES background (e.g., coming from a rich family, living in a big and new house, often buying new things) or a low-SES background (e.g., coming from a poor family, living in a small and old house, rarely buying new things). Each student was described as achieving success in school (i.e., getting one of the highest grades in the class). Each teacher read two vignettes about a low-SES student, and two about a high-SES student. The order of vignettes was randomized.

First, teachers wrote down what they would say in response to the student’s success, if they would say anything. Based on this response, we coded their inflated and modest praise^16^. Second, teachers wrote down why they thought the student achieved the success. Based on this response, we coded their effort, ability, and other attributions^22^. The study materials, data, and analysis code are available via OSF at: https://osf.io/wka4c/.

We tested two preregistered hypotheses. First, we hypothesized that teachers would give low-SES students more praise than high-SES students. Second, we hypothesized that this effect would be stronger for inflated praise than for modest praise. Specifically, we hypothesized that teachers would give low SES students more inflated praise (but not necessarily more modest praise) than high-SES students. We preregistered hypotheses for teacher praise but not for teacher attributions, because there was a good deal of empirical evidence for the positive feedback bias, which provided indirect evidence for our preregistered hypotheses. By contrast, there was no existing empirical evidence to support our hypothesis that students’ socioeconomic status would influence teachers’ attributions.

For each participant, we calculated a praise and attribution difference score (e.g., a positive inflated praise difference score indicates that a teacher praised low-SES students more than high-SES students). We then conducted a one-tailed Wilcoxon signed rank test, with *α* = 0.05, to test whether the location of the distributions of the difference scores was greater than zero.

Of all the responses teachers provided, 84% contained praised. Of all the praise teachers gave, 18% was inflated: 14% for high-SES students and 23% for low-SES students. Thus, low-SES students received more than 1.6 times as much inflated praise as did high-SES students. Most teachers gave low-SES students as much praise as they gave high-SES students. Of all teachers, 18% gave low-SES students more inflated praise than they gave high-SES students. By contrast, only 4% gave low-SES students less inflated praise than they gave high-SES students.

#### Preregistered analyses

Without distinguishing between inflated and modest praise, the location of teachers’ total praise difference scores was not significantly greater than 0, *V* = 87, *p* = 0.644. Thus, overall, teachers did not praise low-SES students more than high-SES students (Supplementary Table 1). Importantly, however, the location of teachers’ inflated praise difference scores was significantly greater than 0, *V* = 240, *p* < 0.001. As hypothesized, teachers gave low-SES students more inflated praise than they gave high-SES students. By contrast, the location of teachers’ modest praise difference scores was not significantly greater than 0, *V* = 71, *p* = 0.997. An exploratory, not-preregistered one-tailed Wilcoxon signed rank test showed that the location of teachers’ modest praise difference scores was significantly smaller than 0, *V* = 71, *p* = 0.003, indicating that teachers gave low-SES students less modest praise than they gave high-SES students. In addition, the location of the difference between the inflated and modest praise difference scores was significantly >0, *V* = 465, *p* < 0.001. Thus, students’ SES had a stronger effect on teachers’ inflated praise than on their modest praise.

Because the study was conducted online, we included a manipulation check to ensure the validity of participants’ responses. After each vignette, participants indicated whether the hypothetical student’s family has a lot or little money. Two participants failed one or more manipulation checks. A sensitivity analysis excluding these participants did not alter the results of our preregistered analyses (i.e., no significant effect became nonsignificant, and no nonsignificant effect became significant).

#### Exploratory analyses

We conducted exploratory analyses. First, we used a two-tailed Wilcoxon signed rank test, with *α* = 0.05, to examine whether teachers were more inclined to attribute a low-SES (vs. high-SES) student’s success to effort. The location of teachers’ effort attribution difference scores was significantly >0, *V* = 338, *p* = 0.002, indicating that teachers attributed the success of low-SES students more often to effort than the success of high-SES students. The location of teachers’ ability attribution difference scores did not significantly deviate from 0, *V* = 101, *p* = 0.878, indicating that teachers attributed the success of low- and high-SES students equally often to ability. The location of teachers’ other attribution difference scores was significantly <0, *V* = 34, *p* < 0.001, indicating that teachers attributed the success of low-SES students less often to other causes than the success of high-SES students. These “other” attributions often reflected external causes (e.g., the child’s home environment or support of a parent or tutor).

Second, we investigated whether teachers with low subjective social status would show less SES biases in praise and attributions (Supplementary Note 1 and Supplementary Tables 1–3). Teachers with low subjective social status praised low- and high-SES students equally often; this was the case for inflated praise, modest praise, and all praise combined. They also attributed the success of low-SES students equally often to effort as they did the success of high-SES students. By contrast, teachers with high subjective social status gave low-SES students more inflated praise and less modest praise than they gave high-SES students. They also attributed the success of low-SES students more often to effort than they did the success of high-SES students. Thus, teachers with low subjective social status showed weaker SES biases in praise and attributions than did teachers with high subjective social status.

#### Summary

Study 1 shows that, on average, teachers indeed gave low-SES students more inflated praise than they gave equally performing high-SES students. Our first hypothesis was rejected: Overall, teachers did not praise low-SES students more often than they praised high-SES students. Our second hypothesis was supported: Teachers gave low-SES students more inflated praise (but not more modest praise) than they gave high-SES students. Why did teachers do so? Our exploratory analyses reveal a possible explanation: Teachers were more inclined to attribute low-SES (vs. high-SES) students’ success to effort. In addition, our exploratory analyses showed that the inclination to attribute low-SES students’ success to effort and to give them inflated praise was less pronounced for teachers with low subjective social status.

### Study 2

Study 2 investigated whether children interpret teachers’ inflated praise as evidence of low ability. The study had a within-subjects experimental design, was administered using paper and pencil, and was conducted during school hours in regular classrooms. Participants were 63 primary school children (ages 10–13), who received informed parental consent. Children 12 or older also provided their own informed consent in addition to informed parental consent. Children read three vignettes in which two students achieved the same outcome (i.e., getting 7 out of 10 questions right) but the teacher decided to praise the students differently. The teacher gave (1) one student modest praise (“You did well!”) and the other student no praise; (2) one student inflated praise (“You did INCREDIBLY well!”) and the other student no praise; or (3) one student inflated praise and the other modest praise. The order of vignettes was randomized. After each vignette, children indicated which student they deemed smarter and which they thought had worked harder. The study materials, data, and analysis code are available via OSF at: https://osf.io/rk9q2/.

We tested three preregistered hypotheses. First, we hypothesized that children would perceive the student who receives modest praise as less smart than the student who receives no praise at all. Second, we hypothesized that children would perceive the student who receives inflated praise as less smart than the student who receives no praise at all. Third, and most importantly, we hypothesized that children would perceive the student who receives inflated praise as less smart than the student who receives modest praise. We preregistered hypotheses for whether children would perceive the student who receives praise as less smart, but not for whether children would perceive the student who receives praise as more hardworking, because there was more extensive existing empirical evidence to support the former than the latter.

#### Preregistered analyses

We used a one-tailed *z*-test, with *α* = 0.05, to test whether more than half (i.e., a proportion of 0.50) of the children deemed one student smarter than the other. The results support our hypotheses. Significantly more than half of the children, *p̂* = 0.76, deemed the student who received modest praise less smart than the student who received no praise, *z* = 4.84, *p* < 0.001. Significantly more than half of the children, *p̂* = 0.76, deemed the student who received inflated praise less smart than the student who received no praise, *z* = 4.84, *p* < 0.001. Significantly more than half of the children, *p̂* = 0.79, deemed the student who received inflated praise less smart than the student who received modest praise, *z* = 5.71, *p* < 0.001.

#### Exploratory analyses

We conducted exploratory analyses. First, we used a one-tailed *z*-test, with *α* = 0.05, to examine whether children thought that one student put in more effort than the other. Significantly more than half of the children, *p̂* = 0.73, thought the student who received modest praise put in more effort than the student who received no praise, *z* = 4.08, *p* < 0.001. Significantly more than half of the children, *p̂* = 73, thought the student who received inflated praise put in more effort than the student who received no praise, *z* = 4.08, *p* < 0.001. Significantly more than half of the children, *p̂* = 0.83, thought the student who received inflated praise put in more effort than the student who received modest praise, *z* = 6.33, *p* < 0.001.

Second, we examined whether children’s gender, age, or subjective social status moderated the effect of praise on which student they deemed smarter or harder working (Supplementary Table 4). There were no significant interactions between praise and gender, age, or subjective social status, underlining the robustness of our findings.

Third, we asked children why they thought the teacher decided to praise one child more than the other. We inspected their open-ended responses. Children suggested that the praised student worked harder, for example: “Because [the non-praised student] knew everything already and [the praised student] still struggled but practiced a lot” and “Because [the praised student] has more difficulties with the topic of that exam and he tried really hard on the exam”. Additionally, children suggested the praised student performed better than usual, for example: “Because [the non-praised student] perhaps gets good grades more often and [the praised student] does not” and “Because [the praised student] normally performs less well.”

#### Summary

Study 2 supports our hypotheses. Children deemed the student who received praise less smart than the student who did not receive praise, regardless of whether the praise was modest or inflated. In addition, children deemed the student who received inflated praise less smart than the student who received modest praise. Why? Exploratory analyses show that children inferred that the student receiving inflated praise worked harder to achieve the same outcome, suggesting that the student had to compensate for low ability.

## Discussion

With two preregistered experiments, we investigated whether inflated teacher praise can make low-SES children seem less smart—but more hardworking. In Study 1, we found that teachers gave low-SES students more inflated praise than they gave high-SES students, even though the students’ success was identical. In addition, they attributed the success of low-SES students more to effort than they did the success of high-SES students. This suggests that teachers gave low-SES students more inflated praise because they believed these students had to work extraordinarily hard to achieve success. In Study 2, we found that teachers’ inflated praise made children seem less smart but more hardworking. Specifically, children deemed a student who received inflated praise from the teacher less smart but more hardworking than a student who received modest praise or no praise at all. These effects were similar for children with high and low subjective social status, suggesting tentatively that children from low-SES backgrounds were as susceptible to these inferences as were their high-SES peers. Together, these findings suggest that teachers’ well-intentioned practices may sometimes backfire and make children from disadvantaged groups seem less competent.

Our research suggests that teachers’ well-intentioned practices may, in some cases, reinforce socioeconomic inequalities in the classroom. Doing so, our research bridges two separate literatures. One literature shows that teachers hold SES biases about children’s intellectual ability^27^. Teachers tend to underestimate the intellectual abilities of low-SES students^31^, hold low expectations for their future success^45^, give them lower grades^46^, and assign them to lower educational tracks^47^. Another literature shows that some well-intentioned teacher practices can signal low ability. For example, when teachers believe a student has low ability, they may be more likely to provide unsolicited help, express pity, and provide comfort-oriented feedback^48–50^. Students readily pick up on these messages, seeing them as evidence that they lack ability^21,51^. By bridging these literatures, the current research suggests that teachers may inadvertently maintain inequality in the classroom by providing inflated praise to low-SES students, making these students seem less smart to their peers. Our research contributes to the broader literature showing that teachers may, unknowingly and unintentionally, reinforce existing inequalities^52^.

Our research contributes to literature on the positive feedback bias. Until now, research in this area has focused predominantly on race and ethnicity^33,38–40^. Our research extends this work to SES and inflated praise, and probes underlying mechanisms. While previous research has uncovered various motives that lead White majority teachers to provide more positive feedback to ethnic minority students (e.g., the motive to see themselves as egalitarian^35^), our research identifies effort attributions as a possible mechanism. When teachers learned that a low-SES student achieved success, they were more inclined to provide this student with inflated praise, and this may have been driven by teachers’ inference that the student’s success stemmed from extraordinary effort. As such, our work integrates literatures on positive feedback bias^33^, inflated praise^53^, and attribution theory^21^ to show that teacher attributions are critical to understanding their inequality-reinforcing practices.

Our findings have implications for understanding the developmental emergence of intellectual stereotypes of low-SES individuals. From the age of 6, children see low-SES individuals as less competent in the academic domain (e.g., less smart) than high-SES individuals^54–57^. Although the early emergence of these stereotypes has been well documented^58^ the mechanisms through which children acquire these stereotypes have not^59^. Children form stereotypes based on implicit environmental cues^60^ and verbal and non-verbal behaviors of others^61^. Our study adds to this research by uncovering the possibility that children learn to view low-SES students as less smart through teachers’ inflated praise. When children observe students belonging to certain social groups (e.g., low SES) consistently receiving more inflated praise from teachers for equal performance, they may infer that the group overall has low intellectual ability. Future research should test this directly.

Our exploratory analyses revealed that teachers with lower subjective social status showed weaker SES biases. They were less inclined to attribute low-SES children’s successes to hard work and less inclined to lavish them with inflated praise. This concurs with research showing that, although teachers generally hold lower expectations for low- than high-SES students, this tendency is weakened among teachers who are themselves from low-SES backgrounds^62^. One possible explanation is that individuals from lower-SES backgrounds, who tend to have lower subjective social status^63^, have experienced more prejudice, stereotyping, and discrimination. Consequently, they may be more aware of societal forces that systematically advantage some social groups and disadvantage others^64^, reducing their tendency to associate SES with intrinsic factors (e.g., effort, ability).

Although not the central focus of our research, we also examined teacher attributions that did not reflect ability or effort (i.e., “other” attributions). These attributions often reflected external causes. Teachers attributed the success of low-SES (vs. high-SES) students less often to other causes, probably because teachers knew that low-SES families have less financial, material, and cultural capital to support their children’s education^65^. Thus, when a low-SES student succeeds despite this relative lack of external support, teachers may be more inclined to attribute the success to internal (rather than external) causes.

How are our findings related to ability and effort praise? Effort praise refers to positive evaluations of children’s effort (e.g., “You must have worked hard at these problems”), whereas ability praise refers to positive evaluations of children’s ability (e.g., “You must be smart at these problems”). Effort praise is generally seen as beneficial to young children’s motivation to learn, because it can communicate that effort is a path to improving ability (i.e., growth mindset)^66^. Yet, to older children (from about age 10), effort praise can communicate that they had to work hard because of low ability^67^. While effort praise communicates this information explicitly, our findings suggest that inflated praise communicates it implicitly. That is, even though the inflated praise made no mention of effort, children readily inferred that those who received inflated praise were more hardworking but less smart. Thus, to prevent older children from making such ability-denigrating inferences, teachers may phrase their praise modestly and focus the praise on children’s strategies rather than their effort (e.g., “You found a good way to do this”).

From an applied perspective, our work underlines the urgency of preventing SES biases in teachers. Our findings suggest three possible solutions. First, in our study, teachers with low subjective social status did not show SES biases in attributions or praise. One solution could therefore be to recruit more teachers from low-SES backgrounds. Providing indirect evidence for this idea, Black students performed better when matched to a Black teacher^68^, particularly in schools with high overall levels of teacher diversity^69^. Second, interventions can help disconnect teachers’ practices from negative stereotypes about low-SES children. Most teachers are motivated to reduce inequality^70^. However, simply making them aware of their negative stereotypes is unlikely to be effective at changing their behavior^71^. An alternative approach is to *sideline bias*, that is, to create situations in which bias is not functional for the goals teachers pursue^72^. For example, research shows that teachers show less bias against low-SES students in contexts emphasizing *learning*—helping all students learn and grow—as opposed to those emphasizing *selection*—identifying and rewarding the smartest students^73^. Third, praise interventions can help teachers provide praise in less biased ways. To date, most praise interventions have focused on increasing teachers’ overall praise^13,74,75^. However, a small-scale study shows that teachers praise more equitably if they monitor their own practices and solicit feedback from colleagues^76^.

Our study has several strengths, including its experimental designs, its inclusion of both teachers and children, and its embrace of open science practices (e.g., preregistration, open materials, open data). Our study also has several limitations. First, we used vignettes to ensure experimental control. In our studies, effects were probably larger than they would be in real life. For example, in Study 1, we presented teachers with information about students’ SES backgrounds. By providing little individuating information about the students themselves (e.g., their personality), we might have increased teachers’ reliance on stereotypes of low- and high-SES students^77^. By providing only information about students’ external socioeconomic conditions (e.g., their house), we might have made teachers more aware of structural barriers faced by low-SES students, encouraging them to attribute low-SES students’ successes to effort. Future studies should investigate inflated praise in teacher-student interactions in real-life classrooms or simulated classrooms in virtual reality^78^. Second, in Study 1, the inter-rater reliabilities of praise and attributions were satisfactory but not excellent. Coders were trained using a set of hypothetical responses (listed in the coding protocol on OSF), which lacked some of the nuances and ambiguities in teachers’ actual responses. To increase reliability in future work, we encourage training with a subset of actual responses.

Third, our study focused on children ages 10–12, who tend to see ability and effort as compensatory^22^. However, there is emerging evidence that even younger children, from about age 4, may see ability and effort as compensatory^79^. This suggests that the adverse effects of inflated praise may already manifest in preschool. Future research should examine this possibility. Fourth, in Study 1, we had teachers read multiple vignettes about high- and low-SES students, so that we could estimate SES biases per teacher, increasing statistical power. We used this within-subjects experimental design successfully before^80^. A limitation is that vignettes were highly similar, except for the information they provided about students’ SES. As a result, teachers may have felt demotivated and suspected our interest in SES. To address this concern, we took steps to minimize potential biases. We randomized the order of vignettes to reduce order effects, and we examined subtle distinctions between modest and inflated praise to minimize demand effects. Yet, we call for well-powered between-subjects experiments to replicate our findings.

Our findings pave the way for future work on teacher feedback and achievement inequality. First, researchers could examine how teachers’ inflated praise influences the self-views of its recipient. Low-SES children tend to have low self-perceived ability and low self-esteem^27,81,82^. Could teachers’ inflated praise contribute to these socioeconomic disparities in children’s self-views? And could these disparities in children’s self-views, in turn, contribute to achievement inequality? Research provides suggestive evidence, indicating that inflated praise can make children feel less competent and worthy^17^, and that these self-views can, in turn, undermine academic achievement^83^. Second, researchers will do well to examine how teachers respond to high- and low-SES children’s failures. Consistent with the present findings, recent work indicates that adults attribute low-SES children’s successes more to effort than to intelligence, whereas they attribute low-SES children’s failures more to a lack of intelligence than a lack of effort^80^. Such low-ability attributions of failure could inspire pity. Teachers feel more pity for children with low ability^48,84^. When teachers pity children, they may comfort them for low ability—“Don’t worry, not everyone can be good at math”^49^. Pity can undercut children’s self-views. In one experiment, when teachers showed pity, children inferred they lacked ability, felt less competent, lowered their expectations, and persisted less^85^. Future work should examine teachers’ pity and comfort-oriented feedback in response to the failure of high- and low-SES children.

Third, researchers could examine cross-cultural differences in the consequences of inflated praise. We conducted our studies in the Netherlands, a prototypically individualistic country where teachers praise their students frequently^86^. In countries where teachers praise more selectively, such as China or Japan, students may draw even more extreme inferences from inflated praise. Indeed, when teachers praise more selectively, children consider the praise more informative^87^. Fourth, researchers should examine intersectionality. Our work focuses on SES, while previous research on teacher feedback has focused primarily on ethnicity and race. Unfortunately, little research has examined intersectionality—the consequences of multiple intersecting social identities^88^. Would low-SES children with multiple stigmatized or minoritized identities be more likely to face biased treatment by teachers? Or would they be less likely to face such treatment, as they might be seen as a less prototypical member of the low-SES group, rendering them “invisible”^89^? Addressing these questions will uncover the consequences of SES biases in teaching practices.

Achievement inequality is a pressing societal problem. Teachers could inadvertently reinforce existing inequalities by praising low-SES children in inflated ways. In our preregistered experiments, teachers gave low-SES students more inflated praise than they gave high-SES students, and children interpreted inflated praise as a sign of low ability. Together, these findings show that teachers may engage in practices that, although well-intentioned, could reinforce negative stereotypes about the intellectual abilities of low-SES children.

## Methods

### Study 1

#### Participants

We conducted a power analysis for a Wilcoxon signed rank test using G*Power^90^. The goal was to detect an effect size of Cohen’s *D*_z_ = 0.25, with *α* = 0.05. We set this effect size because our work was inspired by the positive feedback bias. Prior work^39^ has demonstrated a positive feedback bias in European teachers of Cohen’s *d* = 0.20. Because we differentiated between inflated and modest praise, we expected a slightly larger effect in our study. The test was one-tailed, because our hypotheses are directional. To achieve a power of 0.80, the required sample size was *N* = 106 teachers. We therefore preregistered a sample size of 106 teachers.

Participants were recruited via a professional teacher association, school boards, personal networks, and social media. Participants who reached the end of the questionnaire and taught in primary education were eligible. Of the 313 participants who read at least one vignette, 143 participants were eligible. As preregistered, we included the first *N* = 106 eligible participants (91.51% women, 7.55% men, 0.94% other gender) ages 21–63 (*M* = 38.36 years, *SD* = 11.96) with 0–49 years of experience (*M* = 12.91, SD = 10.62). Five teachers were born and worked in Belgium; all other teachers were born and worked in the Netherlands. The study was approved by the Ethics Review Board of the Faculty of Social and Behavioural Sciences at the University of Amsterdam (2022-CDE-14372). In line with the protocol approved by the Ethics Review Board, participants read an information letter and consent form prior to participation, and they gave their informed consent by actively clicking to the next page (i.e., starting the experiment). Because all data were collected anonymously, we did not collect informed consent in writing, as this would include participants’ names or signatures.

#### Procedure

**Fig. 1:**
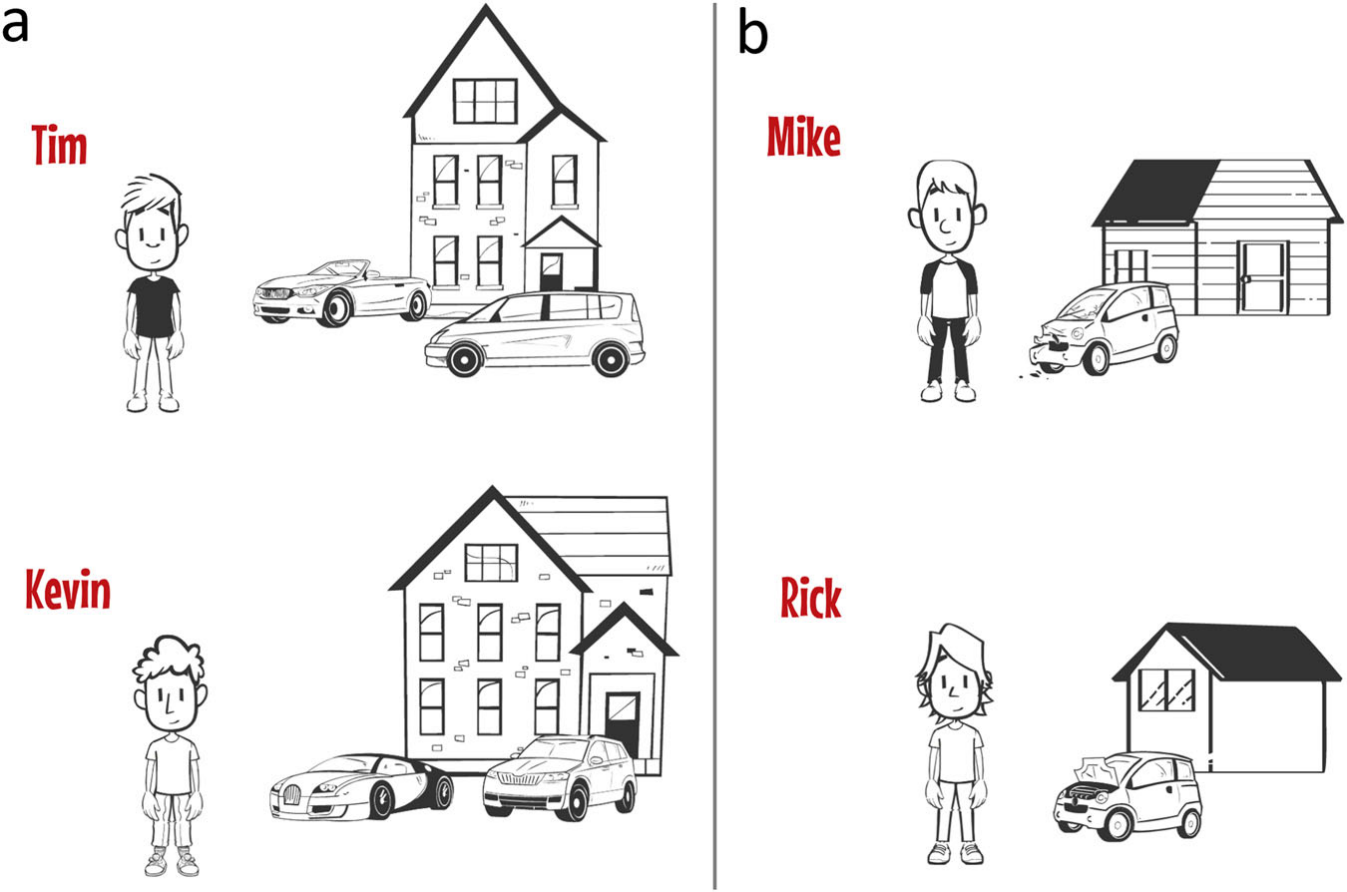
Examples of illustrations of high-SES and low-SES students used in Study 1. **a** Shows high-SES students. **b** Shows low-SES students.

We administered the study online. Participants read four vignettes presented in random order. We adapted the vignettes from prior work^91^, and we used them successfully before^80^. Each vignette described a hypothetical 11-year-old student who has a success experience in school (for the vignettes and illustrations, see Table 1 and Fig. 1, respectively). Two vignettes described a student from a high-SES background, and two described a student from a low-SES background. Consistent with prior work^91^, we manipulated students’ SES by varying information about their external socioeconomic conditions (rather than their internal qualities, such as traits and preferences). For this reason, most text in the vignettes was devoted to describing these conditions.

**Table 1:** Vignettes of academic success of high-SES and low-SES students used in Study 1.

Intro	You are going to read about a child named Tim. Imagine that you’re Tim’s teacher. Tim is 11 years old.
High SES	Tim’s family is rich and has a lot of money. The house where Tim lives is big and new. He lives there with his parents, brothers, and sisters. You can see that it is a very big house. It has 12 rooms and a big swimming pool in the garden. The items in the house are new. His family has two cars: one sports car and another big new car. Tim’s family has a lot of money, so he can buy a lot of new things. His backpack and shoes are new and expensive. His parents have a lot of money, so they often go on trips and vacation with Tim. Tim’s family has enough money to buy all the food they want, so Tim eats a lot of tasty and healthy things.
Low SES	Tim’s family is poor and has very little money. The house where Tim lives is small and old. He lives there with his parents, brothers, and sisters. You can see that it is a very small house. The things in the house are old and worn out. His family has one small car. It is old and often cannot start. Tim’s family has little money, so he cannot buy a lot of new things. His backpack and shoes are old and worn out. His parents also do not have enough money to go on trips or vacations with Tim. Sometimes Tim’s family doesn’t have enough money to buy all the food they want, so Tim eats few tasty or healthy things.
Success	Recently, you administered an important exam in Tim’s class. Tim and his classmates all took the same exam. Tim did very well on the exam. He got one of the highest grades of the class.
Response question	Imagine that you are sharing the grades with the students in the class. What would you say to Tim at that moment? Write down exactly what you would say to Tim, word for word. [open ended]
Attribution question	Why did Tim perform so well on the exam, do you think? Write down what you think, even if you are not sure. Rely on your intuition. There is no right or wrong. [open ended]

Each participants read four vignettes (two high-SES, two low-SES). The gender of the protagonist was matched to the participant’s self-reported gender (man, woman, gender neutral). The order of vignettes was randomized. “Tim” is used as an example here. In the actual vignettes, the names of the protagonist were randomized within genders (men: Rick, Tim, Mike, Kevin; women: Laura, Kim, Lisa, Sanne; gender neutral: Nicky, Guus, Sasha, Robin).

The high-SES student was described as coming from a rich family, living in a big and new house, having two cars, often buying new things, often going on trips and holidays, and having the money to buy tasty and healthy foods. The student was depicted in front of a big house and two cars. By contrast, the low-SES student was described as coming from a poor family, living in a small and old house, having one old and broken-down car, rarely buying new things, rarely going on trips or holidays, and not having the money to buy tasty or healthy foods. The student was depicted in front of a small house and one broken-down car. We presented these two pieces of information (i.e., house, car) because prior research shows that children and adults readily use such cues to evaluate a person’s social standing and resources^56,92^. Then, the vignettes described the student’s success experience (i.e., getting one of the highest grades in the class), which was identical across all vignettes. We selected this success experience because it was (a) social comparative (because this would encourage teachers to think about potential differences between students based on students’ SES) and (b) not too exceptional (because teachers might attribute exceptional success, such as winning a prestigious math competition, primarily to ability).

To minimize the possibility of gender-based ingroup-outgroup biases in teachers’ perceptions of students^93^, we matched the vignettes to participants’ self-reported gender, so that women read about girls, men read about boys, and teachers who did not identify as man or woman read about gender-neutral characters (in our sample, one teacher did not identify as man or woman). The content of the vignettes was identical within SES categories. To make sure that participant perceived the hypothetical students as unique individuals, we gave each a different name, a different physical appearance, and different house and car(s) (Fig. 1). We selected names that are common in both high- and low-SES families^94^ and physical appearances that do not reveal SES^95^, so that they could be used for both high- and low-SES vignettes. For each gender, we created two versions of the vignette, so that the names and physical appearances of the high-SES students in one version corresponded to the names and physical appearances of the low-SES students in another version, and vice versa, thereby ruling out any systematic influence of names and physical appearances. Although the size of the house and the number of cars were identical within SES categories, we created two slightly different illustrations of the house and car(s) for each high- or low-SES vignette. After reading each vignette, using open-ended response formats, participants (1) described how they would respond to the student’s success (i.e., what they would say, if they would say something) and (2) provided their attribution(s) of the student’s success (i.e., why they thought the student achieved this success). We used open-ended response formats, without providing participants with any example attributions, so that we would not prime them with possible attributions. Participants took an average of 16 min to complete the survey.

#### Primary measures

Participants’ open-ended responses were coded by two independent coders, who were blind to condition assignment (i.e., they did not know whether the response was to a high- or low-SES student). The original Dutch protocol and its English translation are available on OSF.

##### Praise

We coded the frequency of modest and inflated praise. Praise was defined as positive evaluations of the student’s traits, actions, or products^96^. Inflated praise was defined as praise containing an adverb (e.g., very, incredibly) or adjective (e.g., amazing, fantastic) signaling a very positive evaluation^16^. Examples include: “Your score is incredible” and “Incredibly well done.” All other praise was coded as modest (i.e., non-inflated). Examples include: “That is an okay score” and “Well done.” Examples of vague responses that we judged not to be praise include: “Keep at it” and “I’d give her a high-five.” These examples do not contain a positive evaluation of the student, which is a defining feature of praise^53,96^. Some teachers wrote down multiple instances of praise in response to a single vignette. Thus, praise frequencies could exceed 1. For example, “You did very well! Well done!” has a praise frequency of 2 (1 inflated praise, 1 modest praise). Praise occurrences in a single response were considered distinct if they evaluated a different trait, action, or product or if they were uttered in different sentences. For example, “You did well and are an incredibly smart student!” has a praise frequency of 2 (1 inflated praise, 1 modest praise), because the two evaluations pertain to different traits and actions. By contrast, “You did incredibly well and very well!” has a praise frequency of 1 (1 inflated praise, 0 modest praise), because the two evaluations pertain to the same action and were uttered in the same sentence. Inter-rater reliability for coding the praise (i.e., total praise, regardless of praise type) was acceptable-to-good (*κ* = 0.74) and comparable to the reliabilities for modest praise (*κ* = 0.73) and inflated praise (*κ* = 0.78)^97^. Disagreements among the coders were resolved through discussion.

##### Attributions

We coded the frequency of ability, effort, and other attributions. Ability attributions reflect causes of success based on the students’ aptitude or acquired skill, such as knowledge and skill^22^. Example responses include: “Because Kim is an intelligent girl” and “She masters the material” (with the latter being coded as an ability attribution because it reflects an aptitude or skill—not the process of acquiring this aptitude or skill, such as studying, practicing, or learning). Effort attributions reflect causes of success based on the student’s temporary or sustained effort, such as studying, practicing, and being hardworking^22^. Example responses include: “He studied well” and “She is a hard worker”. All other causes were considered other attributions. Responses coded as other attributions included: “Good home situation” and “Because she received a lot of support and help at home.”

Some teachers provided multiple attributions in response to a single vignette. Thus, attribution frequencies could exceed 1. For example, “Because she received a lot of support and has probably studied hard” contains one other attribution, one effort attribution, and no ability attribution. Attributions were considered distinct if they attributed success to a different cause or were uttered in a different sentence. For example, “Because she received a lot of support and help at home” contains one other attribution (although “support” and “help” may seem like different attributions, they reflect the same cause of success and were uttered in the same sentence). The inter-rater reliability was acceptable-to-good for ability (*κ* = 0.69), effort (*κ* = 0.63), and other attributions (*κ* = 0.70)^97^. Disagreements among the coders were resolved through discussion.

#### Secondary measures

For exploratory analyses, we measured teachers’ subjective social status using the MacArthur scale of subjective social status^98,99^. We used the version that can be used across the lifespan^100^, so as to ensure comparability across Study 1 and 2. Prior to the experiment, teachers were shown a ladder with 10 rungs representing people with different levels of education, income, and occupational prestige. Participants selected the rung where they felt they stood (*M* = 7.24, SD = 1.08). The bottom rung represented the lowest subjective social status and the top rung the highest subjective social status. Each rung had a number, from 1 to 10, with higher numbers indicating higher rungs (so “10” indicated the highest rung).

Although primary school teachers in the Netherlands tend to have a similar educational level, income, and occupational prestige, we still expected sufficient individual differences in subjective social status. Often, individuals base their subjective social status on comparisons with their local environments (e.g., neighborhoods)^101,102^. Due to these comparisons, some teachers may feel higher or lower in social status than would be warranted based on their objective standing.

#### Preregistered analyses

Per participant, we calculated the average praise and attribution frequencies across the two low-SES vignettes and across the two high-SES vignettes (Tables 2 and 3, respectively). We then calculated difference scores for praise and attribution frequencies by subtracting the frequency of the high-SES vignettes from the low-SES vignettes (Supplementary Table 1 and 2, respectively). This resulted in (1) separate praise difference scores for modest praise, inflated praise, and total praise, and (2) separate attribution difference scores for ability, effort, and other attributions. A positive inflated praise difference score, for example, indicates that a teacher praised low-SES students more than high-SES students. A positive effort attribution difference score, for example, indicates that a teacher attributed the success of low-SES students more to effort than the successes of high-SES students.

**Table 2:** Frequencies of average praise scores across high- and low-SES vignettes in Study 1.

Praise score							
	0	0.5	1	1.5	2	2.5	3
Modest praise							
Low SES	26	8	50	5	15	0	2
High SES	23	5	51	4	18	3	2
Inflated praise							
Low SES	72	15	17	0	2	0	0
High SES	83	10	13	0	0	0	0
Total praise							
Low SES	16	0	55	5	25	3	2
High SES	16	2	50	7	26	3	2

There were two low-SES and two high-SES vignettes. Thus, the praise score reflects the average praise frequency across the two vignettes per SES category (e.g., an inflated praise score of 2 for low SES means that the participant provided an average of two instances of inflated praise per low-SES vignette).

**Table 3:** Frequencies of average attribution scores across high- and low-SES vignettes in Study 1.

Attribution score									
	0	0.5	1	1.5	2	2.5	3	4	5
Ability attribution									
Low SES	43	4	46	1	10	0	2	0	0
High SES	41	9	42	3	8	1	2	0	0
Effort attribution									
Low SES	35	6	37	7	17	1	3	0	0
High SES	44	8	32	4	14	1	3	0	0
Other attributions									
Low SES	80	4	12	4	4	2	0	0	0
High SES	60	7	16	7	7	3	0	2	1

There were two low-SES and two high-SES vignettes. Thus, the attribution score reflects the average attribution frequency across the two vignettes per SES category (e.g., an effort attribution score of 2 for low SES means that the participant made an average of two effort attributions per low-SES vignette).

We used a one-tailed Wilcoxon signed rank test, with *α* = 0.05, to test whether the location of the distributions of the praise difference scores was greater than zero. A location significantly greater than zero indicates that teachers praised low-SES students more than high-SES students. The Wilcoxon signed rank test accommodates for the non-normality of our data (normality was rejected for all outcome measures using the Shapiro-Wilk’s test, with *α* = 0.05) by only assuming a symmetric distribution. The praise difference scores were distributed symmetrically around zero. We performed four tests: (1) one compared the total praise difference score against zero; (2) one compared the inflated praise difference score against zero; (3) one compared the modest praise difference score against zero; and (4) one compared the difference between the inflated praise difference score and the modest praise difference score against zero.

### Study 2

#### Participants

We conducted a power analysis for a z-test using G*Power^90^. Per vignette, our main dependent variable was the proportion of participants who deemed one student to be less smart than the other student. If praise has no effect on inferences about ability, the proportion should be ~50% for each vignette. Therefore, the null hypothesis of the z-tests is *p* = 0.50. Our goal was to detect a small-to-medium effect size of Cohen’s *h* = 0.40, with *α* = 0.05. This is equivalent to 70% of the participants seeing one student as less smart than the other student. This estimate was based on previous research showing that, in late childhood, 76% of children see a praised student as less smart than a non-praised student^42^. To be conservative, we based our power analysis on a slightly smaller effect size in our study. The tests were one-tailed, because our hypotheses are directional. To achieve a power of 0.80, the required sample size was *N* = 74 children.

As preregistered, because we were not able to recruit the desired number of 74 participants by June 17, 2022, we ran our analyses with the participations we have tested by that date (i.e., *N* = 63). We did not inspect or analyze the data before terminating data collection. Participants were *N* = 63 children (46.03% boys, 49.21% girls, 4.76% other gender) ages 10–13 (*M* = 11.11 years, *SD* = 0.90). Children were recruited via a primary school in the Netherlands. The study was approved by the Ethics Review Board of the Faculty of Social and Behavioural Sciences at the University of Amsterdam (2022-CDE-14610). In line with the protocol approved by the Ethics Review Board, children’s parents read an information letter and consent form, and they could withdraw their child from the study within two weeks prior to participation by handing in a written consent withdrawal form. No parent withdrew their consent. Children 12 or older also read an information letter and consent form prior to participation, and they gave their informed consent by actively turning the page (i.e., starting the experiment). Because all data were collected anonymously, we did not collect informed consent in writing, as this would include participants’ names or signatures.

#### Procedure

**Fig. 2:**
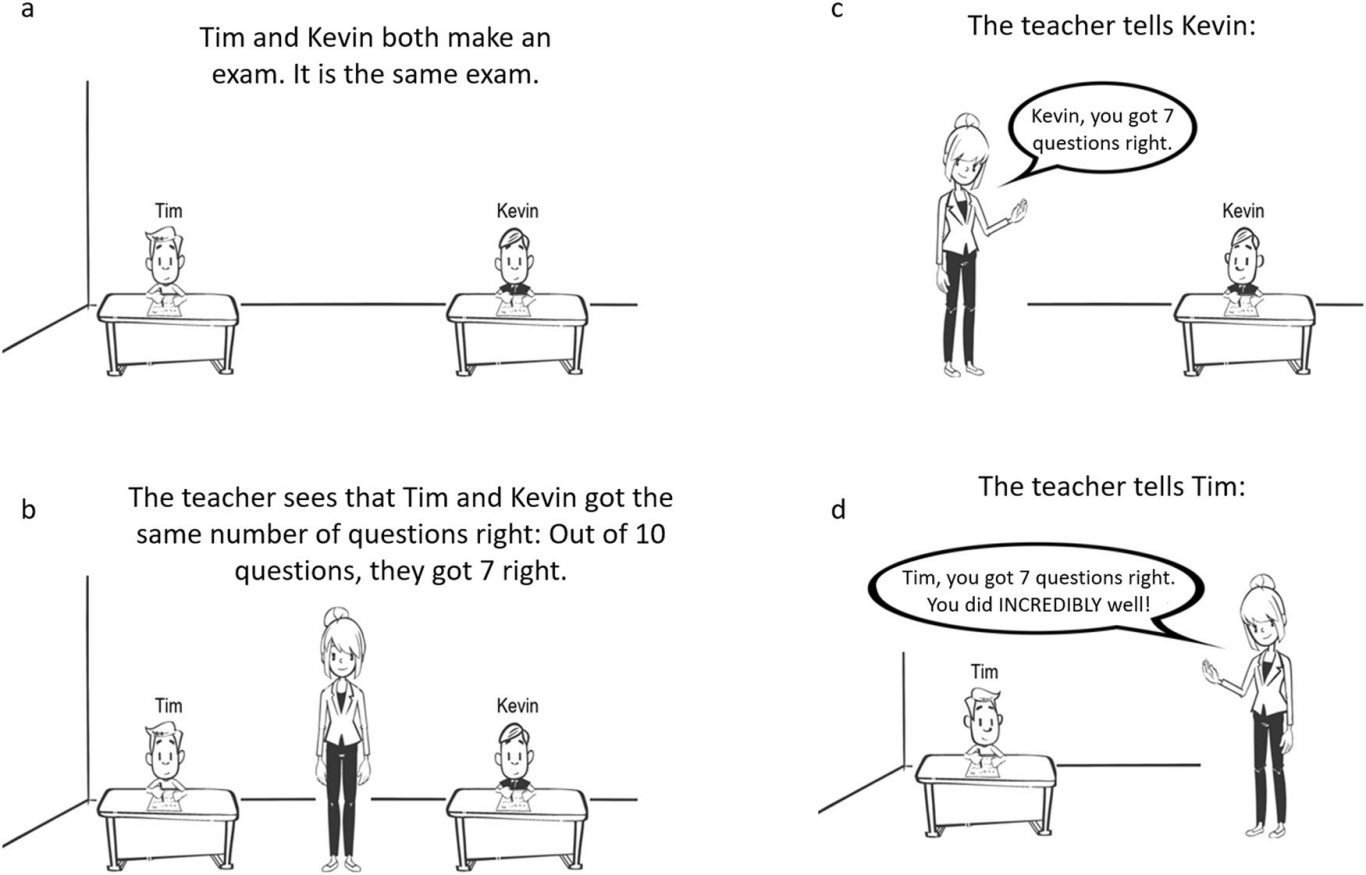
Example of vignettes used in Study 2. This example represents the condition in which one student received inflated praise and the other student received no praise. **a** Shows the students making the exam. **b** Shows the teacher observing that the students got the same number of questions right. **c** Shows one student receiving no praise. **d** Shows the other student receiving inflated praise.

All children read the same three vignettes, presented in the form of an illustration that contained text (Table 4 and Fig. 2). Following prior work^41^, the students depicted in the vignettes were boys and the teacher was a woman. The students had names that are common in both high- and low-SES families^94^. We randomized which student (i.e., the student displayed on the left or displayed in the right) received the more positive praise. We also randomized the order in which the praise conditions were presented. To ensure comparability of Study 1 and 2, we used illustrations that were identical in style to those used in Study 1.

**Table 4:** Vignettes manipulating teacher praise in response to student’s success used in Study 2.

Scene	Translation
1	Tim and Kevin both make an exam. It is the same exam.
2	The teacher sees that Tim and Kevin got the same number of questions right: Out of 10 questions, they got 7 right.
3a	The teacher tells Kevin: “Kevin, you got 7 questions right.”
3b	The teacher tells Kevin: “Kevin, you got 7 questions right. You did well!”
4a	The teacher tells Tim: “Tim, you got 7 questions right. You did well!”
4b	The teacher tells Tim: “Tim, you got 7 questions right. You did INCREDIBLY well!”
5	Tick your answer. Choose one answer per question. 1. Who is the smartest, do you think? [forced choice: Tim, Kevin] 2. Who put in most effort during the exam, do you think? [forced choice: Tim, Kevin] 3a. Why did the teacher praise Tim but not Kevin, do you think? [open ended] 3b. Why did the teacher give more positive praise to Tim than to Kevin, do you think? [open ended]

Each participants read three vignettes. We randomized which student (i.e., the student on the left or right) received modest or inflated praise and we randomized the order in which the praise conditions were presented. “Kevin” and “Tim” are used as examples here. In the actual vignettes, the names of the protagonist were randomized (Tim, Kevin, Rick, Mike, Mark, and Nick).

In the vignette, two students are making the same exam. The teacher observes that the students got the same number of questions right (7 out of 10). Teacher then provides both students neutral feedback: “You got 7 out of 10 questions right.” The neutral feedback was followed by inflated praise (“You did INCREDIBLY well!”), modest praise (“You did well!”), or no praise. There were three conditions: (1) one student received modest praise and the other student received no praise; (2) one student received inflated praise and the other student received no praise; and (3) one student received modest praise and the other student received inflated praise (Table 4 and Fig. 2 for the vignettes).

#### Primary measures

After each vignette, the children were asked: “Who is the smartest, do you think?” and “Who put in most effort during the exam, do you think?” (forced-choice questions, where children could choose between the two students). Children indicated their answer by ticking a box under the face and name of the student of their choice. Additionally, children described why they thought the teacher praised the students differently using an open-ended response format. The vignettes and survey were printed on paper. Children participated during school hours, within their regular classrooms, and took approximately 20 min.

#### Secondary measures

For exploratory analyses, we measured children’s subjective social status using the MacArthur scale of subjective social status^98,99^, the same scale we used in Study 1. Prior to the experiment, children were shown a ladder with 10 rungs representing people with different levels of education, income, and occupational prestige. Children selected the rung where they felt their family stood. The bottom rung represented the lowest subjective social status and the top rung the highest subjective social status. Each rung had a number, from 1 to 10, with higher numbers indicating lower rungs (so “1” indicated the highest rung). Due to an oversight, higher rungs had higher numbers in Study 1 (with “10” indicating the highest rung) but lower numbers in Study 2 (with “1” indicating the highest rung). Importantly, in both studies, our instructions to participants were in line with the numbers they were presented, so participants interpreted the rungs correctly. We reverse-coded the values in Study 2 so that higher numbers indicate a higher subjective social status (*M* = 7.21, SD = 1.20), as in Study 1.

#### Preregistered analyses

Each vignette contained one student who was praised more than the other student (i.e., the student who received inflated praise while the other received no praise or modest praise, or the student who received modest praise while the other received no praise). For each vignette, we calculated the proportion of children who deemed this student to be less smart than the other student and the proportion of children who deemed this student to have worked harder than the other student.

We used a one-tailed *z*-test, with *α* = 0.05, to test whether more than half (i.e., proportion of 0.50) of the children inferred that the student who was praised more was less smart. We ran a *z*-test for each of our three hypotheses: (1) one compared the proportion of children who indicated that the student receiving modest praise is less smart than the student receiving no praise; (2) one compared the proportion of children who indicated that the student receiving inflated praise is less smart than the student receiving no praise; and (3) one compared the proportion of children who indicated that the student receiving inflated praise is less smart than the student receiving modest praise. A hypothesis was supported when the proportion was significantly >0.50.

### Reporting summary

Further information on research design is available in the Nature Research Reporting Summary linked to this article.

### Supplementary information

Supplementary Material

Reporting Summary

## Data Availability

The Study 1 data are available via OSF at: https://osf.io/wka4c/. The Study 2 data are available via OSF at: https://osf.io/rk9q2/.

## Code availability

The Study 1 analysis code is available via OSF at: https://osf.io/wka4c/. The Study 2 analysis code is available via OSF at: https://osf.io/rk9q2/.
