# Supplementary material for: “You did incredibly well!”: teachers’ inflated praise can make children from low-SES backgrounds seem less smart (but more hardworking)
Source: NPJ Sci Learn. 2023 Sep 1;8:31. doi: 10.1038/s41539-023-00183-w (PMC10474104; doi:10.1038/s41539-023-00183-w)
Supplement: Supplementary file 1 — Supplementary Material [file 41539_2023_183_MOESM1_ESM.pdf]

Supplementary Note 1

As exploratory analyses, we investigated whether teachers with low subjective social status would show less SES biases in praise and attributions. To minimize false positives, we conducted these analyses using two-tailed tests, and we adjusted the α level using a Bonferroni adjustment to correct for multiple families of tests.

Study 1

We investigated whether teachers with low subjective social status attributed the success of low- and high-SES students differently than did teachers with high subjective social status. We repeated the analyses of teachers' praise and attributions for teachers with low subjective social status (who reported a subjective social status up to and including the median of ; N_{lowSSS} = 60) and teachers with high subjective social status (who reported a subjective social status above the median of 7; N_{highSSS} = 46). We performed a Wilcoxon signed rank tests, with a Bonferroni-adjusted α = .025. See Tables 3 and 4 for descriptive statistics and Supplementary Table 3 for all analyses.

Praise. Without distinguishing between inflated and modest praise, teachers with high subjective social status praised low- and high-SES students equally often (V = 10.5, p = .588). Importantly, however, they gave low-SES students more inflated praise (V = 66, p = .003) and less modest praise (V = 13, p = .006) than high-SES students. By contrast, teachers with low subjective social status praised low and high SES students equally often regardless of praise type.

Attributions. Teachers with high subjective social status attributed the success of low-SES students more often to effort than the success of high-SES students (V = 114, p = .015). By contrast, teachers with low subjective social status attributed successes of low and high SES students equally often to effort (V = 64, p = .051). Teachers with low (V = 8, p = .047) and high subjective social status (V = 47, p = .044) both attributed the success of low and high SES students equally often to ability. Teachers with low (V = 19, p = .002) and high subjective social status (V = 0, p < .001) both attributed successes of low SES students less often to other causes than the successes of high SES students.

Study 2

We investigated whether children's ability and effort attributions were dependent on their gender (male, female), age (10, 11, 12), or subjective social status (high, low) using Chi-Squared tests, with a Bonferroni-adjusted $\alpha = .017$. For the analyses of subjective social status, we identified children with low subjective social status (who reported a subjective social status up to and including the median of 7; $N_{lowSSS} = 49$) and children with high subjective social status (who reported a subjective social status above the median of 7; $N_{highSSS} = 12$). No tests were significant (Supplementary Table 4). The ability and effort estimations were not significantly dependent on gender, age, or subjective social status across praise conditions.

Supplementary Tables

Supplementary Table 1

Frequencies of Praise Difference Scores in Study 1

	Praise difference score							
	-1.5	-1	-0.5	0	0.5	1	1.5	2
All teachers (*N* = 106)								
Modest	0	8	14	80	1	3	0	0
Inflated	0	0	4	83	13	6	0	0
Total	0	1	10	87	6	2	0	0
Inflated - modest	1	2	4	73	10	9	1	6
Teachers with low subjective social status (*N* = 60)								
Modest	0	3	5	49	1	2	0	0
Inflated	0	0	4	48	6	2	0	0
Total	0	1	5	48	5	1	0	0
Teachers with high subjective social status (*N* = 46)								
Modest	0	5	9	31	0	1	0	0
Inflated	0	0	0	35	7	4	0	0
Total	0	0	5	39	1	1	0	0

Note. Praise difference scores reflect the average frequency of praise provided to low-SES students minus the average frequency of praise provided to high-SES students. A higher praise difference score indicates that the participant gave more praise to low-SES than high-SES students (e.g., an inflated praise score of 1 means that the participant provided, on average, one instance of inflated praise more to a low-SES student than to a high-SES student).

Supplementary Table 2

Frequencies of Attribution Difference Scores in Study 1

	Attribution difference score												
	-4	-3.5	-3	-2.5	-2	-1.5	-1	-0.5	0	0.5	1	1.5	2
	All teachers (*N* = 106)												
Ability	0	0	0	0	0	0	4	7	86	5	3	0	1
Effort	0	0	0	0	0	0	1	6	78	9	8	4	0
Other	1	0	1	3	3	4	11	10	71	0	2	0	0
	Teachers with low subjective social status (*N* = 60)												
Ability	0	0	0	0	0	0	4	5	50	0	1	0	0
Effort	0	0	0	0	0	0	1	2	48	3	4	2	0
Other	0	0	1	1	1	3	6	5	41	0	2	0	0
	Teachers with high subjective social status (*N* = 46)												
Ability	0	0	0	0	0	0	0	2	36	5	2	0	1
Effort	0	0	0	0	0	0	0	4	30	6	4	2	0
Other	1	0	0	2	2	1	5	5	30	0	0	0	0

Note. Attribution difference scores reflect the average frequency of attributions made in response to low-SES students minus the average frequency of attributions made in response to high-SES students. A higher attribution difference score indicates that the participant made more attributions in response to low-SES than high-SES students (e.g., an effort attribution score of 1 means that the participant made, on average, one effort attribution more in response a low-SES than a high-SES student).

Supplementary Table 3

Wilcoxon Signed-Rank Test for Praise and Attribution Difference Scores for Teachers With Low and High Subjective Social Status in Study 1

	Teachers with low subjective social status		Teachers with high subjective social status	
	V	*p*	*V*	*p*
Praise difference score				
Modest	22	.315	13	.006*
Inflated	56	.166	66	.003*
Total	39	> .999	11	.588
Attribution difference score				
Ability	8	.047	47	.044
Effort	64	.051	114	.015*
Other	19	.002*	0	< .001*

*$p < .025$

Supplementary Table 4

Chi-Square Examining Whether Attributions Differed by Gender, Age, and Subjective Social Status in Study 2

	Gender			Age			Subjective Social Status		
	χ^2	*df*	*p*	χ^2	*df*	*p*	χ^2	*df*	*p*
Ability Attributions									
No praise vs Modest praise	0.17	2	.915	2.79	3	.425	3.36	1	.067
No praise vs Inflated praise	1.19	2	.551	0.80	3	.848	< .01	1	1.00[a]
Modest praise vs Inflated praise	1.49	2	.475	3.26	3	.354	< .01	1	.964
Effort Attributions									
No praise vs Modest praise	1.08	2	.582	1.30	3	.730	< .01	1	1.00[a]
No praise vs Inflated praise	0.09	2	.957	4.37	3	.224	< .01	1	1.00[a]
Modest praise vs Inflated praise	0.87	2	.647	1.99	3	.574	0.31	1	.578

[a]Rounded up to 1.00

Supplementary References

1. Adler, N. E., Epel, E. S., Castellazzo, G. & Ickovics, J. R. Relationship of subjective and objective social status with psychological and physiological functioning: Preliminary data in healthy white women. *Health Psychol.* **19**, 586–592 (2000).
2. Goodman, E. *et al.* Adolescents' perceptions of social status: development and evaluation of a new indicator. *Pediatrics* **108**, e31–e31 (2001).
3. Amir, D., Valeggia, C., Srinivasan, M., Sugiyama, L. S. & Dunham, Y. Measuring subjective social status in children of diverse societies. *PLoS One* **14**, e0226550 (2019).
4. Mistry, R. S., Brown, C. S., White, E. S., Chow, K. A. & Gillen-O'Neel, C. Elementary school children's reasoning about social class: A mixed-methods study. *Child Dev.* **86**, 1653–1671 (2015).
5. Peretz-Lange, R., Harvey, T. & Blake, P. R. From "haves" to "have nots": Developmental declines in subjective social status reflect children's growing consideration of what they do not have. *Cognition* **223**, 105027 (2022).
6. Singh-Manoux, A., Adler, N. E. & Marmot, M. G. Subjective social status: Its determinants and its association with measures of ill-health in the whitehall ii study. *Soc. Sci. Med.* **56**, 1321–1333 (2003).
7. Kang, S. J. & Seo, W. Do people determine their subjective socioeconomic status based on the housing type and residential neighborhood? Empirical evidence from seoul. *Land* **11**, 2036 (2022).
