# Supplementary material for: “You did incredibly well!”: teachers’ inflated praise can make children from low-SES backgrounds seem less smart (but more hardworking)
Source: NPJ Sci Learn. 2023 Sep 1;8:31. doi: 10.1038/s41539-023-00183-w (PMC10474104; doi:10.1038/s41539-023-00183-w)
Supplement: Supplementary file 2 — Reporting Summary [file 41539_2023_183_MOESM2_ESM.pdf]

nature portfolio

Corresponding author(s): Emiel Schoneveld & Eddie Brummelman

Last updated by author(s): May 4, 2023

Reporting Summary

Nature Portfolio wishes to improve the reproducibility of the work that we publish. This form provides structure for consistency and transparency in reporting. For further information on Nature Portfolio policies, see our Editorial Policies and the Editorial Policy Checklist.

Statistics

For all statistical analyses, confirm that the following items are present in the figure legend, table legend, main text, or Methods section.

n/a	Confirmed	
☐	☒	The exact sample size (*n*) for each experimental group/condition, given as a discrete number and unit of measurement
☐	☒	A statement on whether measurements were taken from distinct samples or whether the same sample was measured repeatedly
☐	☒	The statistical test(s) used AND whether they are one- or two-sided<br>*Only common tests should be described solely by name; describe more complex techniques in the Methods section.*
☐	☒	A description of all covariates tested
☐	☒	A description of any assumptions or corrections, such as tests of normality and adjustment for multiple comparisons
☐	☒	A full description of the statistical parameters including central tendency (e.g. means) or other basic estimates (e.g. regression coefficient) AND variation (e.g. standard deviation) or associated estimates of uncertainty (e.g. confidence intervals)
☐	☒	For null hypothesis testing, the test statistic (e.g. F, t, r) with confidence intervals, effect sizes, degrees of freedom and P value noted<br>*Give P values as exact values whenever suitable.*
☒	☐	For Bayesian analysis, information on the choice of priors and Markov chain Monte Carlo settings
☒	☐	For hierarchical and complex designs, identification of the appropriate level for tests and full reporting of outcomes
☐	☒	Estimates of effect sizes (e.g. Cohen's d, Pearson's r), indicating how they were calculated

Our web collection on statistics for biologists contains articles on many of the points above.

Software and code

Policy information about availability of computer code

Data collection	The data of Study 1 was collected using an online survey in the commercial software Qualtrics. All materials are available via OSF at: https://osf.io/wka4c/<br><br>The data of Study 2 was collected using a paper-pencil questionnaire. The vignettes and randomized versions of the questionnaire were made using custom python code. All materials and code are available via OSF at: https://osf.io/rk9q2/
Data analysis	All analyses were performed in R. The code used for the analyses of Study 1 is available via OSF at: https://osf.io/wka4c/<br>The code used for the analyses of Study 2 is available via OSF at: https://osf.io/rk9q2/

For manuscripts utilizing custom algorithms or software that are central to the research but not yet described in published literature, software must be made available to editors and reviewers. We strongly encourage code deposition in a community repository (e.g. GitHub). See the Nature Portfolio guidelines for submitting code & software for further information.

Data

Policy information about availability of data

All manuscripts must include a data availability statement. This statement should provide the following information, where applicable:

- Accession codes, unique identifiers, or web links for publicly available datasets
- A description of any restrictions on data availability
- For clinical datasets or third party data, please ensure that the statement adheres to our policy

All data are avalaible via OSF. The data of Study 1 are available at: https://osf.io/wka4c/
The data of Study 2 are available at: https://osf.io/rk9q2/

Research involving human participants, their data, or biological material

Policy information about studies with human participants or human data. See also policy information about sex, gender (identity/presentation), and sexual orientation and race, ethnicity and racism.

Reporting on sex and gender	We assessed gender via self-report in both Study 1 and Study 2. Study 1 included 106 participants (91% women, 8% men, 1% other gender). Study 2 included 63 participants (46% boys, 49% girls, 5% other gender).
Reporting on race, ethnicity, or other socially relevant groupings	In Study 1, we assessed country of birth and country of employment via self-report. Of 106 participants, 101 were born and worked in the Netherlands; 5 were born and worked in Belgium. In both Study 1 and Study 2, we assessed participants' subjective social status via self-report.
Population characteristics	In Study 1, participants were primary-school teachers. In Study 2, participants were primary-school children
Recruitment	In Study 1, participants were recruited via a professional teacher association, school boards, personal networks, and social media. In Study 2, participants were recruited via a primary school in the Netherlands.
Ethics oversight	Ethics Review Board of the Faculty of Social and Behavioural Sciences at the University of Amsterdam (Study 1: 2022-CDE-14372; Study 2: 2022-CDE-14610).

Note that full information on the approval of the study protocol must also be provided in the manuscript.

Field-specific reporting

Please select the one below that is the best fit for your research. If you are not sure, read the appropriate sections before making your selection.

☐ Life sciences ☒ Behavioural & social sciences ☐ Ecological, evolutionary & environmental sciences

For a reference copy of the document with all sections, see nature.com/documents/nr-reporting-summary-flat.pdf

Behavioural & social sciences study design

All studies must disclose on these points even when the disclosure is negative.

Study description	We conducted two preregistered experiments. We used hypothetical scenarios to ensure experimental control. In Study 1, teachers complete an online survey in which they responded to the successes of hypothetical students from low- and high-SES backgrounds. We coded the frequency of praise in these responses. This frequency data was used to investigate whether teachers provided more praise to low- than high-SES students. In Study 2, children read vignettes in which a teacher praised two hypothetical students differently for the same success. Children indicated which student they deemed smarter and more hardworking. This data was used to investigate how children interpret teacher praise.
Research sample	Study 1: All participants were teachers teaching in Dutch or Belgian primary school (in which students are aged 4 to 12). Teachers' experience ranged from 0 to 49 years ($M = 12.91$, $SD = 10.62$). Study 2: All participants were children were aged 10-13 years ($M = 11.11$, $SD = 0.90$).
Sampling strategy	For Study 1: We conducted a power analysis for a Wilcoxon signed rank test using the program G*Power. The goal was to detect an effect size of Cohen's Dz = .25, with $\alpha = .05$. We set this effect size because our work was inspired by the positive feedback bias. Prior work has demonstrated a positive feedback bias in European teachers of Cohen's = 0.2. Because we differentiated between inflated and modest praise, we expected a slightly larger effect in our study. The test was one-tailed, because our hypotheses are directional. To achieve a power of 0.80, the required sample size was $N = 106$ teachers. We therefore preregistered recruiting 106 teachers. Participants were recruited via a professional teacher association, school boards, personal networks, and social media.<br>For Study 2: We conducted a power analysis for a z-test using the program G*Power. Per vignette, our main dependent variable was the proportion of participants who deemed one student to be less smart than the other student. If praise has no effect on inferences about ability, the proportion should be around 50% for each vignette. Therefore, the null hypothesis of the z-tests is $p = .50$. Our goal was to detect a small-to-medium effect size of Cohen's $h = .4$, with $\alpha = .05$. This is equivalent to 70% of the participants seeing one student as less smart than the other student. This estimate was based on previous research showing that, in late childhood, 76% of

children see a praised student as less smart than a non-praised student. To be conservative, we based our power analysis on a slightly smaller effect size in our study. The tests was one-tailed, because our hypotheses are directional. To achieve a power of .80, the required sample size was N = 74 children.
As preregistered, because we were not able to recruit the desired number of 74 participants by June 17, 2022, we ran our analyses with the participations we have tested by that date (i.e., N = 63). We did not inspect or analyze the data before terminating data collection. Children were recruited via a primary school in the Netherlands.

Data collection	Study 1 was conducted online. Teachers complete an online survey in which they responded to the successes of hypothetical students from low- and high-SES backgrounds. Study 2 was conducted using paper-and-pencil methods in children's regular classrooms. Children read vignettes in which a teacher praised two hypothetical students differently for the same success; they then complete questions about these students.
Timing	Data was collected from April to August 2022
Data exclusions	No data were excluded
Non-participation	In Study 1, participants who reached the end of the questionnaire and taught in primary education were eligible. Of the 313 participants who read at least one vignette, 143 participants were eligible. As preregistered, we included the first N = 106 eligible participants.
Randomization	STUDY 1: Participants read four vignettes presented in random order. Each vignette described a hypothetical 11-year-old student who has a success experience in school. Two vignettes described a student from a high-SES background, and two described a student from a low-SES background. The high-SES student was described as coming from a rich family, living in a big and new house, having two cars, often buying new things, often going on trips and holidays, and having the money to buy tasty and healthy foods. The student was depicted in front of a big house and two cars. By contrast, the low-SES student was described as coming from a poor family, living in a small and old house, having one old and broken-down car, rarely buying new things, rarely going on trips or holidays, and not having the money to buy tasty or healthy foods. The student was depicted in front of a small house and one broken down car. We presented these two pieces of information (i.e., house, car) because prior research shows that children and adults readily use such cues to evaluate a person's social standing and resources. Then, the vignettes described the student's success experience (i.e., getting one of the highest grades of the class), which was identical across all vignettes. We selected this success experience that was (a) social comparative (because this would encourage teachers to think about potential differences between students in their SES) and (b) not too exceptional (because teachers might attribute exceptional success, such as winning a prestigious math competition, primarily to ability).<br>The vignettes were matched to participants' self-reported gender, so that women read about girls, men read about boys, and teachers who did not identify as man or woman read about gender-neutral characters (in our sample, one teacher did not identify as man or woman). The content of the vignettes was identical within SES categories. To make sure that participant perceived the hypothetical students as unique individuals, we gave each a different name, a different physical appearance, and different house and car(s). We selected names that are common in both high- and low-SES families and physical appearances that do not reveal SES, so that they could be used for both high- and low-SES vignettes. For each gender, we created two versions of the vignette, so that the names and physical appearances of the high-SES students in one version corresponded to the names and physical appearances of the low-SES students in another version, and vice versa, thereby ruling out any systematic influence of names and physical appearances. Although the size of the house and the number of cars was identical within SES categories, we created two slightly different illustrations of the house and car(s) for each high- or low-SES vignette. After reading each vignette, using open-ended response formats, participants (1) described how they would respond to the student's success (i.e., what they would say, if they would say something) and (2) provided their attribution(s) of the student's success (i.e., why they thought the student achieved this success). We used open-ended response formats, without providing participants with any example attributions, so that we would not prime them with possible attributions. Participants took an average of 16 minutes to complete the survey.<br><br>STUDY 2: All children read the same three vignettes, presented in the form of an illustration that contained text. Following prior work, the students depicted in the vignettes were boys and the teacher was a woman. The students had names that are common in both high- and low-SES families. We randomized which student (i.e., the student displayed on the left or displayed in the right) received the more positive praise. We also randomized the order in which the praise conditions were presented. To ensure comparability of Study 1 and 2, we used illustrations that were identical in style to those used in Study 1.<br>In the vignette, two students are making the same exam. The teacher observes that they got the same number of questions right (7 out of 10). Teacher provides both students neutral feedback: "You got 7 out of 10 questions right." The neutral feedback was followed by inflated praise ("You did INCREDIBLY well!"), modest praise ("You did well!"), or no praise. There were three different scenarios: (1) one student received modest praise and the other student received no praise; (2) one student received inflated praise and the other student received no praise; and (3) one student received modest praise and the other student received inflated praise.

Reporting for specific materials, systems and methods

We require information from authors about some types of materials, experimental systems and methods used in many studies. Here, indicate whether each material, system or method listed is relevant to your study. If you are not sure if a list item applies to your research, read the appropriate section before selecting a response.

Materials & experimental systems

n/a	Involved in the study
☒	☐ Antibodies
☒	☐ Eukaryotic cell lines
☒	☐ Palaeontology and archaeology
☒	☐ Animals and other organisms
☒	☐ Clinical data
☒	☐ Dual use research of concern
☒	☐ Plants

Methods

n/a	Involved in the study
☒	☐ ChIP-seq
☒	☐ Flow cytometry
☒	☐ MRI-based neuroimaging
